# M. Rostan’s Clinical Lecture

**Published:** 1843-10-14

**Authors:** 

**Affiliations:** Paris


### THE MEDICAL EXAMINER,

### And Retrospect of the Medical Sciences.

Vol. VI.] PHILADELPHIA, SATURDAY, OCTOBER 14, 1843. [No. 20.

### A CLINICAL LECTURE.

### ON THE TREATMENT OF ACUTE AFFECTIONS OF THE BRAIN.

*Delivered at the Hôtel Dieu, Paris.*

BY M. ROSTAN.

From the earliest period this important subject has occupied the attention of physicians; but, as this class of diseases was but little known, as very often the symptoms alone were regarded, and the cause overlooked, it necessarily followed that the means employed to combat them were ordinarily empirical, and often irrational. It is then only since the labours of the moderns, since myself, with Dr. Rochoux, and other observers, have seriously studied this branch of pathology, that the essential and differential characters of the affections to which they referred, have been established in a precise manner, and that their treatment has become logical and rational.

Among the means employed to combat these affections, that which undoubtedly holds the first rank, is bloodletting either general or local. Thus, when we have to treat a cerebral hemorrhage, where it is slight, a treatment almost expectant, such as diluent drinks, diet, &c., will suffice; where it is of medium intensity, and necessarily graver, you must then immediately recur to sanguine emissions, which you can graduate to the forces and age of the patient, and the intensity of the disease. It is difficult, nay, almost impossible to give any accurate rules in this respect; you can only say, that the bleedings should be more abundant in meningitis, or in frank meningo-encephalitis, than in cases of hemorrhage. Experience has placed beyond doubt this principle of therapeutics. Now, where should we practice bleeding? On this point practitioners are not agreed. Some prefer bleeding from the foot; for in this manner, say they, you produce revulsion, and more efficaciously disgorge the cerebral vessels. For me, for a long time, this pretended revulsive efficacy of bloodletting from the foot is pure hypothesis. I have employed it very often simultaneously with venesection from the arm, for the purpose of making a parallel, and I have never been able to recognize its superiority, so that I have adopted as a principle to bleed ordinarily from the arm, and have always found it advantageous.

In very bad cases can we not draw blood more efficaciously from the jugular vein? For a long time this variety of bleeding was acknowledged to be very useful and was generally practised, and I shared the opinion. But, if we consider on one side, that to arrest the bleeding from a large vein which has been opened, you must apply a circular bandage around the neck, quite tight, which may favour the cerebral congestion, in place of resolving it; if you reflect upon the danger of the introduction of air into this vein, and the terrible accidents which result from it, placed beyond all doubt by the multiplied experiments of Dr. Amussat, you will acknowledge that we are right in abandoning it. After repeated general bleedings more or less, according to circumstances, come local bloodletting by means of leeches and cups. To what part of the body should they be applied? On this point the opinion of physicians is divided. Some (and these are the partisans of revulsive bleedings) prefer that they should be applied by preference to parts at a distance from the head, in order that this supposed revulsion may be produced. Myself, with many judicious practitioners, are in the habit of applying them behind the ears; the result is always satisfactory, and much more speedy than a contrary practice. This then is the chief, sovereign medication, upon which more than any other we should rely, in the treatment of these grave affections; this is a remedy, of the efficacy of which no practical physician will now-a-days raise the slightest doubt; for I do not regard as practitioners, physicians who, reasoning after purely physical laws, deny the usefulness of bleeding in this class of affections, and even think it prejudicial, by emptying the vessels of the brain, thus producing a vacuum, which must necessarily be filled by other blood, which would flow thither with violence. These are good theories, which practice by the bed side completely overturns, and which can only come from the heads of physicians who know more of physics than physic.

After bleeding, diluent and refrigerant drinks rank next, in the acute form of the disease. They serve absolutely to dilute the blood, to remove from it that plasticity, that excess of fibrin which renders it more liable to stagnate in the vessels.

In the third place I will mention laxatives and purgatives, which are now-a-days generally employed in this class of affections. These means, independently of the depletion and derivation they exercise on the intestinal canal, as soon as absorbed, produce a general effect of hyposthenia, by introducing into the blood principles far more capable than simple diluent drinks of diminishing the plasticity and fibrinous state of the blood. I believe that the preference should be given to those that act specially on the rectum, and they may be administered either by the mouth or as enemata.

Some physicians have extolled very much the employment of tartar emetic. Portal was a warm partisan of it, and speaks a great deal about it in his works. He says that the vomiting was useful by acting somewhat in the manner of a revulsive. I object to this practice rather as dangerous than as inefficacious; for the efforts made to vomit by patients invite blood to the head, in the place of diverting it, and in this manner augment the hemorrhage, or the existing inflammation. Nevertheless if the stomach is full of food, you may, if not contraindicated, administer an emetic, to clear out that organ, and yet this end is better attained by the administration of a weak infusion of tea or linden, with less risk of the dangers just mentioned.

One of the methods of treatment, which may be called hygienic, and which should never be lost sight of, is the suitable position of the patient. He should be so placed that when in bed, his head is elevated. The advantages of this position are generally well known and appreciated, especially of late years, since the influence of the laws of gravitation on the organism, has been acknowledged. The profound judgment of Bichat comprehended the practical application of physical science, and called the attention of physicians to the subject, and it is since this that it has here been constantly and usefully employed. To the elevated position of the head, should be added, the removal of the hair, if too long; the use of soft cushions filled with oats, so that the head be not too heated, as generally happens with the ordinary pillow.

A powerful accessory are cold applications to the forehead. Practitioners generally use them, and I have always derived advantage from them. You may employ compresses dipped in cold water, or water and vinegar, which must be renewed so soon as they become warm; or pounded ice in a bladder. I prefer, myself, continued irrigation, whenever it can be properly superintended. This method is employed with great success in surgery in cases of complicated fracture in lacerated wounds. The method I employ is the same; it is familiar to you and I will not stop to describe it. Unluckily it is not convenient in hospitals, as it requires constant superintendance; it is necessary that a nurse should be continually by the bed side of the patient, so that the threads are not disturbed, that the water should not cease to flow, and that all inflammatory reaction be foreseen. In private practice perpetual assistance is easy, and it is a means which you should not neglect in cases of this nature.

Finally, revulsives to the extremities constitute good adjuvants to the other methods just indicated.

Such are the means that constitute about the treatment in cerebral hemorrhages of gravity, and the acute period of meningo-encephalitis.

When the disease lasts for some time without resolution, and when it may be regarded as chronic, (the acute stage being subdued,) we must have recourse to other means. These are, powerful cutaneous revulsives, such as blisters, moxas, setons, cauteries, &c., which are applied to different parts of the body, but principally to the nucha behind the ears, upon the temples. These means favour interstitial absorption, and the resolution of what remains of the disease.

What should we think of the action of strychnine in these cerebral affections? It is not long since, when this medicine was regarded as all powerful against all the consecutive phenomena of acute cerebral affections, such as paralysis, etc.

It having been observed, by some physicians, that nux vomica produced spasms, movements more or less energetic in the extremities, when administered, it was thought that when motion was diminished, or lost, that strychnine might restore it. Numerous experiments were made respecting it, and a physiological physician read a very long memoir on the efficacy of this medicine in paralysis, at the Academy of Medicine. Others, wih myself, experimented on the pretended efficacy of this heroic remedy, and it was discovered that its power was very much exaggerated; and this was very easy to understand, even *à priori*, for very often the cause of paralysis, (which is ordinarily a pure and simple effect of an affection of the nervous centres,) has for cause the existence in the brain of some abnormal product, tuberculous, cancerous, &c.; now, these affections cannot be cured by the use of strychnine, which is absolutely incapable of repairing profound organic alterations. Hence my opinion, in regard to it, is decided. Nevertheless I do not refuse to prescribe in cases where all other known means have failed.

Electricity has been employed and praised for some time past, in the treatment of chronic maladies of the nervous system. The same remarks that I have just made regarding strychnine, are equally applicable to it. Its efficacy has been singularly exaggerated.

Thirty years ago Hallè was ordered by the Academy of Sciences, to make a report with one of his colleagues, on the utility of this therapeutic agent in paralysis. These physicians submitted fifty-one paralytics to the effects of electricity. What was the result? Some few were relieved; others derived no marked benefits from it; and others, again, experienced contrary effects. The reporters accordingly concluded that electricity was useful in some cases of paralysis; useless in many; and injurious to others. But they do not determine the different cases, nor establish the destinctive and differential characters of paralysis, because they did not seek the morbid causes. They could not consequently infer any rule for the application of electricity in these cases. Now, we proceed very differently in the diagnosis of this affection, and our local diagnosis is much more exact than that of our predecessors. Hence we know better those cases where electricity is indicated and where it is not, and our therapeutic efforts are more assured.

I will not stop to discuss the mineral waters of Barèges, Plombieres, &c,, which are so recommended to poor paralytics, when all the other resources of our art have been laid under contribution. I believe that the waters themselves can do but little, but they are useful in obliging the patient to change the climate, scene, &c., to travel, which may exercise a salutary influence.

It remains for me now to say a few words on the preservative treatment of this class of affections.

There are many very useful precepts to follow in this respect, but these are not the powders of Dr. Amand, nor the other numerous amulets, the offspring of charlatanism, and sustained by ignorance, which can preserve us from cerebral affections, and especially from those under which we already suffer. Portal proposed a crowd of prophylactics. The best are those furnished us by wise hygienic precautions. Thus, persons who have already suffered, or who are predisposed to these affections, should adhere to a strict regimen; the diet should be vegetable; they should use refrigerant diluent drinks; they should religiously avoid exposure to the heat of a summer's sun, or to intense cold; they should not expose themselves to severe mental emotions of any kind; severe and prolonged mental exercise should be shunned; and finally, they should sleep with the head uncovered, and on hard pillows.

*Paris, August*, 1843.

